# The potential threat of *Strongyloides* spp. to mountain gorillas and public health in Bwindi Impenetrable National Park, Uganda

**DOI:** 10.1007/s00436-026-08643-z

**Published:** 2026-02-18

**Authors:** Nelson Bukamba, Kelly Marie Sambucci, Eva Nosková, Vojtech Baláž, Ricky Okwir Okello, Dilly Dan Muhumuza, Fred Nizeyimana, Neil Donald Sargison, Rob Francis Kelly, Benard Ssebide, Tierra Smiley Evans, Kirsten Gilardi, Klára Judita Petrželková, Barbora Pafčo, Barbora Červená

**Affiliations:** 1 Gorilla Doctors, MGVP, Inc., Davis, CA USA; 2 Institute of Vertebrate Biology, Czech Academy of Sciences, Brno, Czech Republic; 3 Department of Anthropology, Faculty of Science, Masaryk University, Brno, Czech Republic; 4 Department of Ecology and Diseases of Zoo Animals, Game, Fish and Bees, Faculty of Veterinary Hygiene and Ecology, University of Veterinary Sciences, Brno, Czech Republic; 5 The Royal (Dick) School of Veterinary Studies, University of Edinburgh, Edinburgh, UK; 6 Department of Integrative Biology and Infectious Diseases and Vaccinology Division, University of California, Berkeley, CA USA; 7 Institute of Parasitology, The Czech Academy of Sciences, České Budějovice, Czech Republic; 8 Liberec Zoo, Liberec, Czech Republic; 9 Department of Pathology and Parasitology, Faculty of Veterinary Sciences, University of Veterinary Sciences Brno, Brno, Czech Republic

**Keywords:** Strongyloides, Public health, Mountain gorilla, Gastrointestinal parasites, Uganda, Zoonoses

## Abstract

Soil-transmitted nematodes *Strongyloides* spp. parasitize a range of vertebrates, including endangered great apes and humans. Since 2018, the Mountain Gorilla Veterinary Project has observed an increase in gastrointestinal parasitic infections, including suspected strongyloidiasis in mountain gorillas in Bwindi Impenetrable National Park (BINP), Uganda. To investigate the prevalence of *Strongyloides* and potential cross-host transmission, we conducted noninvasive fecal sampling of mountain gorillas, olive baboons, domestic dogs and pigs. Samples were examined by coproscopy and real-time PCR followed by sequencing of the HVR-IV 18S rRNA and *cox1* genes. *Strongyloides* prevalence was higher in mountain gorillas ranging near the park edge compared to forest interiors inside the park and among infant and juvenile mountain gorillas compared to other age groups. *Strongyloides fuelleborni*, a known zoonotic species, was identified in both baboons and mountain gorillas, suggesting sharing this particular parasite between the two primate species with potential implications for humans surrounding the park. In domestic animals, *S. ransomi* was detected in pigs, a zoonotic lineage *S. stercoralis* haplotype was detected in one dog, and another haplotype closely related to *S. ransomi* and *S. venezuelensis* was identified in another dog. The presence of zoonotic *S. fuelleborni* and *S. stercoralis* in Bwindi ecosystem poses a threat to mountain gorilla as well as human public health. Our study underscores the need for continued monitoring and community sensitization about the risk of cross-species transmission of these parasites.

**Supplementary Information:**

The online version contains supplementary material available at 10.1007/s00436-026-08643-z.

## Introduction

*Strongyloides* spp. are soil-transmitted rhabditid nematodes that parasitize the gastrointestinal system of a variety of vertebrate hosts with cross-host transmission potential (Thamsborg et al. 2017). Among the ~ 60 known *Strongyloides* species, two – *S. stercoralis* and *S. fuelleborni* – infect humans as well as other primates, including great apes (Nutman 2017; Nosková et al. 2024). Since 2018, veterinarians from the Mountain Gorilla Veterinary Project (MGVP) have identified an increase in morbidity and mortality of mountain gorillas in Bwindi Impenetrable National Park (BINP) from severe gastrointestinal parasitic infections, a condition they have termed as chronic wasting (CW). Clinical symptoms of CW include emaciation, severe diarrhea, anemia, lethargy, browning of the hair coat, stunted growth, and in some instances, death. In several cases of CW in infant gorillas, high numbers (reaching thousands of eggs per gram of feces) of *Strongyloides* eggs have been observed (MGVP veterinary records) suggesting the potential role of these parasites in clinical illness.

Infection with *Strongyloides* spp. in humans ranges from light asymptomatic infections to chronic symptomatic strongyloidiasis and in rare events, particularly among immunocompromised individuals, life-threatening hyperinfection characterized by larval dissemination throughout organ systems (Buonfrate et al. 2013; Nutman 2017). In veterinary medicine, other *Strongyloides* spp. are associated with clinical disease mainly in young animals such as piglets, lambs, calves and foals (Deplazes et al. 2016; Thamsborg et al. 2017). However, captive non-human primates (NHP), including great apes, can develop severe strongyloidiasis from *S. stercoralis* infection with symptoms including acute lethargy, periodic dry cough, bloated abdomen, pneumonia, anemia, vomiting, and acute diarrhea (Leeflang and Markham 1986; Lowenstine et al. 2018). Except for primates, including humans, dogs are known hosts of *S. stercoralis*. Genetic analyses show two lineages, potentially species: lineage A occurring in dogs, humans, and other primates, though the transmission between dogs and humans is rare, and lineage B typically found in dogs (Barratt et al. 2019; Bradbury and Streit 2024; Nosková et al. 2024).

Globally, strongyloidiasis is considered a neglected tropical disease and estimated 300–600 million people are infected, with a high incidence recorded in both tropical and subtropical regions (Buonfrate et al. 2013; Puthiyakunnon et al. 2014; Beknazarova et al. 2016). In Africa, the overall prevalence of human *Strongyloides* infections is estimated to be between < 1% and 1.60% (Odeniran and Ademola 2016; Eslahi et al. 2021), however this is likely an underestimate (Eslahi et al. 2021). Indeed, in Uganda, occasional local coproscopy-based studies have reported *S. stercoralis* prevalence ranging from 0.2% in a refugee camp in Mid-Western Uganda to up to 13% prevalence in fishing villages in Mukono, central Uganda (Lule et al. 2010; Nampijja et al. 2015; Morawski et al. 2017; Sanya et al. 2020). A recent study determined 8% prevalence of *S. stercoralis* in Mukono using multiplex real-time PCR (Natukunda et al. 2024). Finally, both *S. stercoralis* and *S. fuelleborni* have been detected in people living in the Bwindi Impenetrable National Park (Ashford et al. 1990). *Strongyloides* spp. have also been reported in Ugandan domestic animals including *S. avium* in poultry (Ssenyonga 1982), *S. ransomi* in pigs (Roesel et al. 2017; Ngwili et al. 2022), and unidentified *Strongyloides* species in goats (Gradé et al. 2007; Kalule et al. 2023) and various wildlife, such as ungulates (Guilbride et al. 1962; Apio et al. 2006; Fugazzola and Stancampiano 2012) or rodents in BINP (Mawanda et al. 2020). Finally, *Strongyloides* spp. were recorded from multiple NHP species, including mountain gorillas (Ashford et al. 1990; Kalema-Zikusoka et al. 2005) and olive baboons (Hope et al. 2004) in BINP, guenons from western Uganda (Gillespie et al. 2004), chimpanzees in Kibale and Bulindi forests (Muehlenbein 2005; Bezjian et al. 2008; Hasegawa et al. 2016; McLennan et al. 2017), and colobus monkeys in Kibale and Lake Nabugabo (Gillespie et al. 2005; Hodder and Chapman 2012). Of these studies, only Hasegawa et al. (2016) characterized the *Strongyloides* larvae as *S. fuelleborni* by nuclear and mitochondrial markers. Moreover, there are globally only a few epidemiological studies evaluating cross-host transmission of *S. stercoralis* (Hasegawa et al. 2016; Jaleta et al. 2017; Barratt et al. 2019; Beknazarova et al. 2019) and more are needed to determine the likelihood of transmission across different animal taxa.

**Fig. 1:**
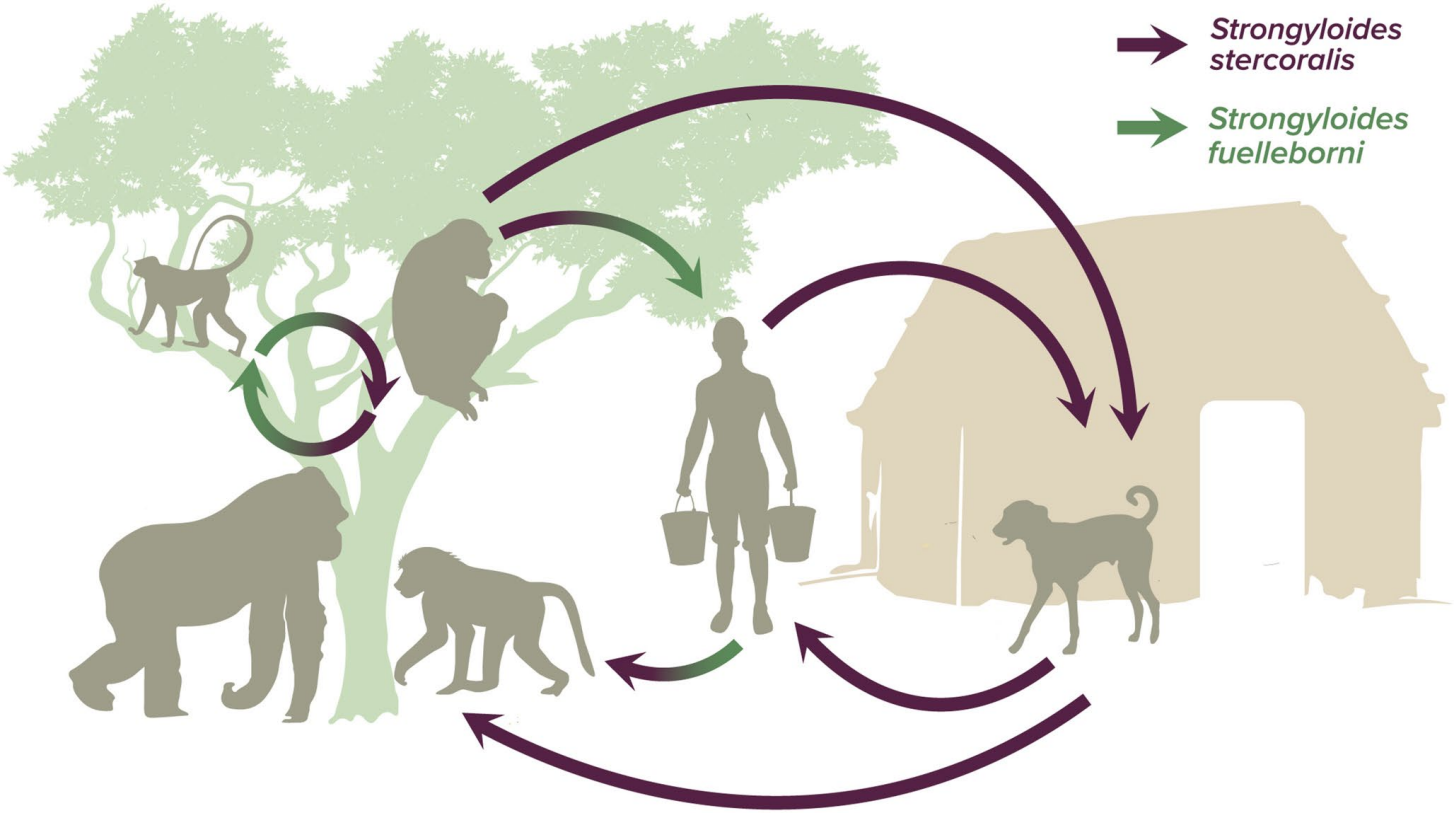
Potential pathways for *Strongyloides* spp. transmission at the domestic-wildlife interface in Bwindi. Both species can circulate among non-human primates inside the forest, but humans can get infected by both species as well, while domestic dogs are known hosts of *S. stercoralis*. Baboons and dogs are often moving between the forest and community land and can easily spread the infectious stages in both environments

The BINP is surrounded by a dense human population living in basic conditions typical for rural areas and some parts of the park have no buffer zones, facilitating interactions between humans and primates crossing the forest edge and park border (McNeilage et al. 2001; Mwima and McNeilage 2003; Seiler and Robbins 2016; Muylaert et al. 2021). Such conditions can enable transmission of pathogens, including *Strongyloides* (Schär et al. 2013; Muylaert et al. 2021) (Fig. 1). Given the potential of *Strongyloides* spp. to be a threat to human public health, as demonstrated by recently emerged clinical cases of strongyloidiasis in Uganda (Pecorella et al. 2022; Yahaya et al. 2023), as well as a threat to the endangered mountain gorillas, the aim of this study was to investigate the prevalence of these nematodes in mountain gorillas and other free-ranging wildlife and domestic animals around BINP. Furthermore, our goal was to evaluate sharing *Strongyloides* spp. among the studied hosts using both coproscopy and molecular techniques, which has not yet been done in this ecosystem.

## Materials and methods

### Sampling and coproscopy

Targeted and opportunistic fecal samples were collected non-invasively from mountain gorillas (*N* = 141), olive baboons (*N* = 27), and dogs (*N* = 25) around BINP between November 2021 and November 2022. Mountain gorilla samples were collected from the night nests after the animals had left. In olive baboons, the animals were followed and observed from a safe distance, and when defecation occurred, a sample was collected after the individuals moved away. Finally, feces from the dogs were collected after spontaneous defecation. Sex and age category of mountain gorillas, together with group affinity, were available for gorilla samples (Schaller 1963; Sinayitutse et al. 2021). Gorilla groups were further categorized based on their ranging patterns: those inhabiting areas near the forest edge and those residing deep within the forest interior. Non-invasively collected (from the pig pens) fecal samples from pigs (*N* = 110) were opportunistically available from another study. In dogs and pigs, samples were collected with informed consent from the owners. Aliquots of fresh fecal samples were preserved in 10% formalin for coproscopic analyses and in 96% ethanol for molecular analyses. Fecal sediment was obtained by homogenizing, sieving and centrifuging (2000 RPM for 3 min) the formalin-preserved samples. Fecal sediment was weighed, and then all resuspended in 10 ml of 10% formalin. Finally, 1 ml of the resuspended fecal sediment was centrifuged (1500 RPM for 2 min), and the total sediment was transferred onto a slide for Strongyloides egg and larvae identification. Strongyloides eggs were quantified and recalculated as eggs per gram of fecal sediment (EPGS). Mountain gorilla samples were examined fresh by Mini-FLOTAC© as described in Petrželková et al. (2021) and quantified as eggs per gram of feces (EPGF).

### Molecular methods

Ethanol preserved samples were used for DNA extraction using the DNeasy^®^ Power Soil Pro Kit (Qiagen, Hilden, Germany) and subsequent detection of *Strongyloides* spp. DNA using probe based real-time PCR (qPCR) (Verweij et al. 2009). A subset of positive samples from each host species was used for 2-step PCR amplification of the hypervariable region IV of the 18S rRNA gene (HVR-IV; ~220 bp) and the cytochrome c oxidase 1 gene (*cox1;* 217 bp) as described by Nosková et al. (2026). Sanger sequencing was performed in both directions (Macrogen Europe B. V., Amsterdam, Netherlands). DNA sequences were manually checked and trimmed in Geneious Prime^®^ 2023.2.1 (www.geneious.com) followed by BLAST analysis. Obtained sequences were aligned with representative *Strongyloides* spp. sequences downloaded from GenBank and the resulting alignments were used to construct maximum likelihood phylogenetic trees by IQ-TREE (Trifinopoulos et al. 2016). The most suitable model was chosen by ModelFinder (Kalyaanamoorthy et al. 2017) implemented in IQ-TREE based on the highest Bayesian information criterion scores and weights. The tree topology was tested by 1000 replicates of ultrafast bootstrapping (Minh et al. 2013) and Shimodaira–Hasegawa (SH)-like approximate likelihood ratio test (Anisimova et al. 2011). The trees were visualized using the iTOL v6 software (Letunic and Bork 2024).

### Statistics

Differences between the prevalence obtained by microscopy and qPCR (method effectiveness) calculated separately in each host were evaluated by McNemar’s test in IBM SPSS Statistics (Version 20). For testing the differences in *Strongyloides *qPCR prevalence between the factors (group, forest position, sex/age category and sex), we used General Linear Models (GLM) with binomial distribution. For sex/age category, Tukey post-hoc tests were employed to test for differences among levels of factorial explanatory variables.

## Results

### Baboons had the highest *Strongyloides* prevalence and fecal sediment egg counts

Eggs corresponding to *Strongyloides* spp. were identified in baboon, pig, and mountain gorilla fecal samples. No eggs were found in dog samples, but a larva with morphology typical for *S. stercoralis* L1 was detected in one dog sample. Other observed gastrointestinal parasites included *Entamoeba* spp., *Trichuris* sp., *Buxtonella*-like ciliates and strongylid nematodes in baboons, *Trichuris* sp., strongylids, *Entamoeba* spp. and *Anoplocephala* sp. in mountain gorillas, *Ancylostoma* sp. in dogs and *Ascaris* sp., *Balantioides* sp., *Eimeria* spp. and *Trichuris* sp. in pigs. The highest *Strongyloides* egg counts and prevalence were observed in baboons compared to other host species (Table 1). Overall, there was a significant difference between the microscopic and qPCR methods, with qPCR detecting more *Strongyloides*-positive samples. The difference between the two methods was statistically significant in all host species except for the olive baboon (Table 1).

**Table 1:** Prevalence and fecal egg counts expressed as eggs per gram (EPG) of fecal sediment (baboons, pigs) or eggs per gram of feces (only in gorillas) together with McNemar’s test comparing coproscopy and qPCR prevalence in different hosts

Hosts (sample size)	Number of positive samples in coproscopy	Mean EPG ± SD	Number of positive samples by qPCR	McNemar’s chi-square	*p* value
Mountain gorilla (141)	3 (2.13%)	19.1 ± 224	70 (49.6%)	21.6	< 0.001
Olive baboon (27)	16 (59.3%)	116 ± 167	23 (85.2%)	3.6	0.06
Pigs (110)	9 (8.18%)	34.4 ± 120	34 (30.9%)	32.3	< 0.001
Dogs (25)	1 (4%)	1 L1	4 (16%)	12.9	0.0003

### Age category and home range location affected *Strongyloides* prevalence

In mountain gorillas, the group affinity affected the qPCR prevalence of *Strongyloides* (GLM *p* < 0.001), and prevalence ranged from 0% in Kyagurilo B group to 100% in the Nkuringo group. Any sex differences in *Strongyloides* prevalence were not observed (GLM *p* = 0.547), but mountain gorillas in younger age groups were significantly more likely to test positive for *Strongyloides* [juveniles (GLM *p* = 0.047), infants (GLM *p* = 0.004)] compared to older gorillas (sub-adults, black-backs, adult females and silver-backs combined) (Table 2). Furthermore, mountain gorillas ranging nearby the forest edge were 2.14 times more likely to test positive for *Strongyloides* compared to gorillas residing deep in the forest interior (GLM *p* = 0.037) (Table 2).

**Table 2:** *Strongyloides* spp. prevalence by qPCR in mountain gorillas by sex/age group and location

Predictors	*n* sampled	*n* positive (%)
Sex/Age		
ADF	51	10 (19.6)
BB	13	7 (53.8)
INF	14	11 (78.6)
JUV	6	4 (66.7)
SAD	4	3 (75.0)
SB	13	4 (30.8)
Location		
Deep forest	42	13 (31.0)
Forest edge	59	26 (44.1)

*ADF* - adult female, *BB* - black back, *INF - *infant, *JUV *- juvenile, *SAD* - sub-adult, *SB -* silverback. In only 101 samples, sex/age category could be determined based on the fecal lobe size (Schaller 1963; Sinayitutse et al. 2021)

## Baboons and gorillas shared *S*. *fuelleborni*

**Fig. 2:**
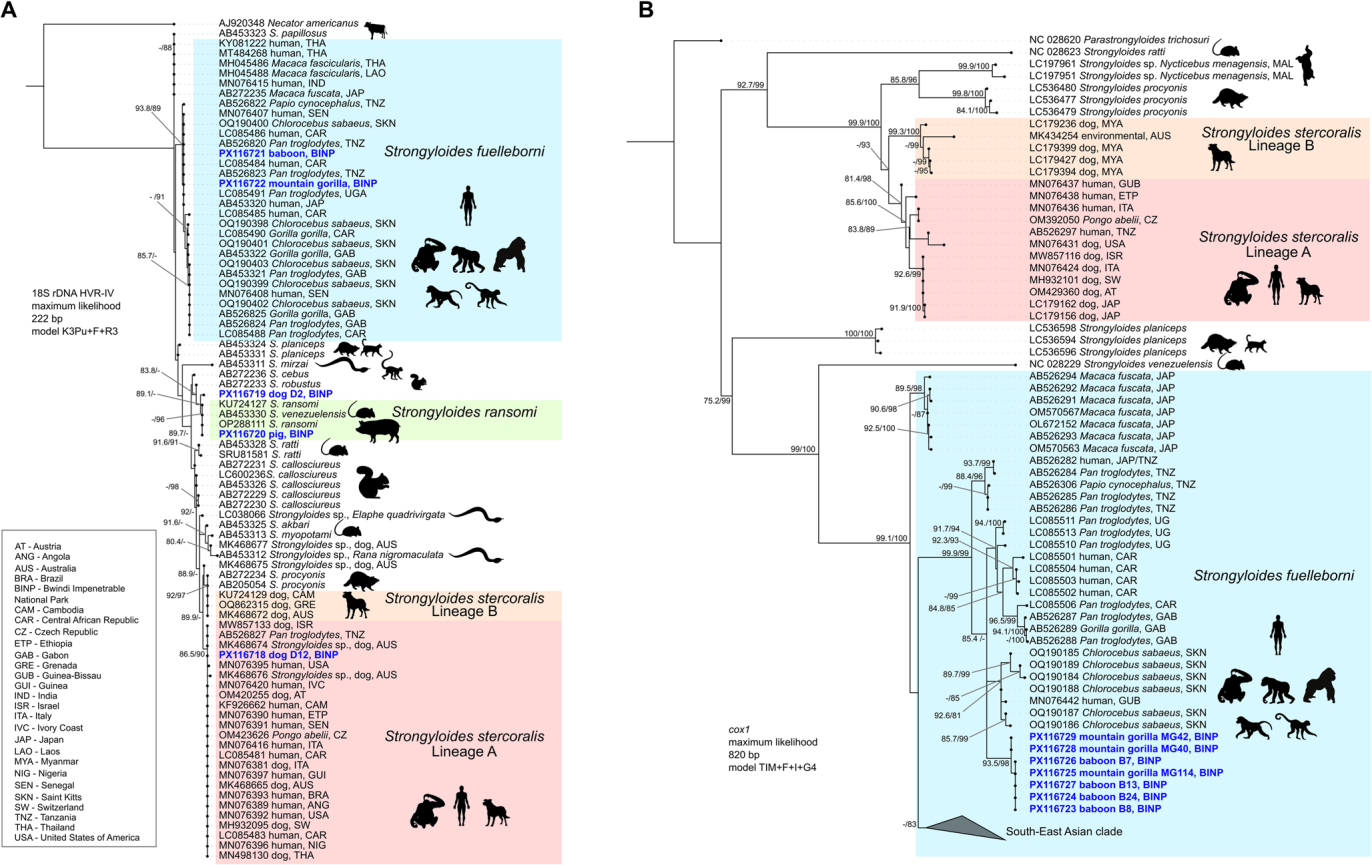
Maximum likelihood phylogenetic tree of *Strongyloides* spp. **a**) 18S rDNA HVR-IV and **b**) *cox1.* Sequences from this study are in blue bold noting the host and code of the sample where relevant. Sequences downloaded from GenBank are labelled by the accession number and where relevant, host and country of origin. For other species, typical hosts are marked by the icons. Numbers at nodes are SH-aLRT support / ultrafast bootstrap support values, both in %. Only values above 80% are shown

Sequencing of HVR-IV was performed on 10 mountain gorilla, 10 baboon, 8 pig, and 4 dog samples that tested positive by qPCR for *Strongyloides* spp. (Supplementary file S1). From the above samples, sequences obtained from gorillas (*n* = 6, GenBank accession no. PX116722) and baboons (*n* = 6, GenBank accession no. PX116721) were identical and showed 100% identity to *S. fuelleborni* isolates from chimpanzees in Bulindi, Uganda (GenBank accession nos. LC085491, LC085492, LC085493, LC085496), and a human in Dzanga Sangha Protected Areas (DSPA), Central African Republic (CAR) (GenBank accession no. LC085484). Three identical sequences of *S. ransomi* (GenBank accession no. PX116720) were obtained from pig samples, while two different haplotypes of *Strongyloides* were obtained from dogs (GenBank accession no. PX116718, PX116719): one showed 100% identity with *S. stercoralis* haplotype E *sensu* Barratt et al. (2019) (GenBank accession no. MK468674; from a dog in Australia) and the other showed 98.6% identity with various *S. ransomi* and *S. venezuelensis* isolates. In the phylogenetic tree (Fig. 2a), where sequences from gorillas, baboons, and pigs were represented by a single consensus sequence, our primate-originating sequences clustered in the African *S. fuelleborni* clade comprising all other sequences from various NHP as well as from humans. The Bwindi pig sequence clustered with other sequences of *S. ransomi* and *S. venezuelensis*. Sister to this clade was the sequence of *Strongyloides* sp. obtained from the dog sample D2, while the other sequence D12 clustered clearly in a *S. stercoralis* clade corresponding to the zoonotic lineage A *sensu* Barratt et al. (2019) comprising sequences from humans, NHP and dogs.

### Lower success in amplifying and sequencing *cox1*

Although HVR-IV was successfully amplified in 17 out of 32 selected samples, *cox1* sequences of good quality were obtained only from 3 gorillas, 4 baboons, 3 pigs and 1 dog sample. Moreover, sequences obtained from pigs and a dog did not belong to any *Strongyloides* spp., but *Ascaris* and *Metastrongylus* in case of pigs or *Ancylostoma caninum* in case of the dog sample were amplified. Five more sequences from gorillas and baboons were of low quality, upon BLAST analysis, one sequence from a gorilla showed high similarity to a *Necator* sp., while the other four to *S. fuelleborni* (**Supplementary file S1**). Although the sequences from gorillas and baboons were of good quality, from 1 to 8 ambiguous nucleotides (double peaks) were detected in each of them, suggesting presence of multiple haplotypes in the sample. When aligned, these heterozygote nucleotides occurred at 16 different positions of the alignment (269 bp). In the phylogenetic tree (Fig. 2b), all the Bwindi baboon and gorilla sequences clustered with other *S. fuelleborni* sequences from Africa, although they formed a separate sub-clade closely related to a clade comprising *S. fuelleborni* sequences from green monkeys in Saint Kitts Island, originally brought to the island from West Africa (Van Der Kuyl and Dekker 1996) and a sequence from a human from Guinea-Bissau (GenBank accession number MN076442).

## Discussion

This study reported the prevalence of *Strongyloides* spp. in multiple hosts sharing the environment based on microscopy combined with molecular methods in the domestic animals and wildlife, including the mountain gorillas living in Bwindi, Uganda, and zoonotic species were identified in several hosts.

Overall, the qPCR detected higher numbers of positive animals compared to microscopy. In baboons, the results between qPCR and microscopy were similar, which is likely due to their comparatively high fecal egg counts. In mountain gorillas, there was a low number of shed eggs, which can be unevenly dispersed in feces and likely contributed to the substantial discrepancy between the number of positive samples detected by microscopy and qPCR. This combined with high numbers of strongylid eggs typically present in the Bwindi gorillas (Petrželková et al. 2021), makes low intensity *Strongyloides* infections more difficult to detect by microscopy, as the *Strongyloides* eggs might be overlooked. On the other hand, the used qPCR assay (Verweij et al. 2009) targets a conservative region of the 18S rDNA thus amplifying multiple *Strongyloides* species. However, as the primers are rather generic, further determination of the detected species is needed to correctly interpret the positive result.

Two commonly employed genetic markers were used to characterize *Strongyloides* spp. around the BINP: HVR-IV of the 18S rDNA and *cox1.* Both have been widely used in humans, NHP and dogs for sequencing of individual larvae or adult worms, and of recent, by metagenomics pipelines (Bradbury et al. 2021). Although the *cox1* gene is widely used as a standard marker for assessment of inter- and intraspecies diversity, and thus potential interspecies transmission, the HVR-IV is also effective for identification of individual *Strongyloides* species (Bradbury et al. 2021; de Ree et al. 2024).

In our study, the amplification and sequencing of the HVR-IV was successful in about half of the samples despite the faint bands in some of them. In contrast, *cox1* showed much lower amplification success rate and sequences contained ambiguous nucleotides suggesting presence of multiple haplotypes within an individual host, which was commonly observed in Australian dogs (Beknazarova et al. 2019) and St. Kitts vervets (Richins et al. 2023). In several cases, sequences were of low quality or other nematodes such as hookworms, lungworms or roundworms, were amplified instead of *Strongyloides.* This is not surprising as Barratt et al. (2019) reported off-target amplification of *Necator* and *Oesophagostomum* in humans using the same primers. On the other hand, the HVR-IV sequences showed only one haplotype in most host species and two haplotypes in dog. These findings are consistent with a previous metagenomic study from humans and dogs reporting multiple HVR-IV haplotypes in only a small percentage of samples (Beknazarova et al. 2019). However, a recent metagenomic study from St. Kitts showed presence of five to six *S. fuelleborni* HVR-IV haplotypes in all analyzed vervet samples (Richins et al. 2023). Therefore, both markers can help with determination of *Strongyloides* species, but the true genetic diversity of *Strongyloides* populations should be evaluated by metagenomic studies which allow for characterization of multiple haplotypes in a single sample.

The use of high-throughput sequencing approaches may also substantially improve the detection of *S. stercoralis*. Although we did not detect any *S. stercoralis* in gorillas or baboons using microscopy or Sanger sequencing, its presence cannot be excluded. The Mini-FLOTAC^©^ method applied to gorilla samples is based on flotation and is therefore unsuitable for detecting *S. stercoralis*, which sheds L1 larvae rather than eggs. While the L1 larvae can, in principle, be detected in the sedimentation method used in baboons, larval stages are not as readily detected as *S. fuelleborni* eggs, particularly in formalin-preserved material where larvae are dead, shed in low numbers, and unevenly distributed (Uparanukraw et al. 1999). Standard microscopy diagnostic methods for *S. stercoralis* rely on active migration of L1 larvae, such as the Baermann larvoscopy or agar plate culture, which are substantially more sensitive than sedimentation techniques (Nosková et al. 2024; Watts et al. 2016). Consequently, failure to detect *S. stercoralis* by microscopy in this study may reflect methodological limitations rather than true absence.

Similarly, low larval output may explain why *S. stercoralis* DNA was not amplified or sequenced. Although both 18S HVR-IV and *cox1* primers used in this study have been previously shown to amplify both *S. stercoralis* and *S. fuelleborni*, amplification efficiency may differ between species. In samples dominated by *S. fuelleborni*, the more abundant taxon is likely to outcompete *S. stercoralis* in the early PCR cycles. As a result, the Sanger sequencing would preferentially recover the dominant taxon and thereby mask low-level *S. stercoralis* infections. High-throughput sequencing would overcome this limitation by enabling detection of low-abundance haplotypes in mixed infections.

Finding *S. fuelleborni* eggs in mountain gorillas and baboons is consistent with previous studies that commonly reported this parasite in the two primate species across BINP, although the prevalence rates differed (Ashford et al. 1996; Hope et al. 2004; Kalema-Zikusoka et al. 2005; Rothman et al. 2006). While the overall microscopy-based prevalence for mountain gorillas found in this study was much lower than reported in the above-mentioned studies, the total qPCR-based prevalence reaching nearly 50%, with some gorilla groups having the prevalence even 100%, was higher than previously reported (Ashford et al. 1990, 1996). Furthermore, Ashford et al. (1996) detected higher *S. fuelleborni* prevalence in young animals which is consistent with our findings. Higher *Strongyloides* prevalence in young animals could be a consequence of the transmammary route of infection from infected breastfeeding mothers as known from dogs (Shoop et al. 2002), although the data on this route of transmission in primates is very scarce (Brown and Girardeau 1977), or to waning maternal immunity in combination with the naivety or immaturity of the infants’ immune system.

On the other hand, while Ashford et al. (1996) and Kalema-Zikusoka et al. (2005) recorded egg counts ranging from 160 to 540 EPG and between 200 and 1200 EPG respectively, the values recorded in our study reached only tens of EPG. This could be a marker of improvements in the health of the general population due to veterinary clinical interventions on targeted individuals deemed fit for deworming under the MGVP clinical intervention policy. This approach has been highlighted by Robbins et al. (2011) as one of the advantages of extreme conservation with respect to endangered mountain gorillas.

Gorilla groups ranging at the edge of the forest and therefore the park border had significantly higher prevalence of *Strongyloides* than the groups residing inside the park. Baboons sampled in this study also live at the very edge of the forest, moving frequently between the community land and the park. These baboons had the highest prevalence, which was higher than previously reported by Hope et al. (2004), as well as the highest fecal egg counts among our studied hosts. The forest edge represents an important environment for pathogen transmission due to increased direct contact between people, their domestic animals and wildlife as well as environmental contamination by animal and human feces. Human legal or illegal activities at the forest edge such as wood collection or wire setting are often accompanied by poor sanitation as many villages close to the park lack permanent latrines (Creel et al. 2002; Chapman et al. 2006). High prevalence of *Strongyloides* spp. in both baboons and mountain gorilla groups ranging at the edge of the forest confirms an increased risk of *Strongyloides* infection in this shared environment. Moreover, detection of the same *S. fuelleborni* haplotype in both primate species suggests an ongoing cross-host transmission.

Several *Strongyloides* spp. have been detected in domestic dogs and pigs. Unsurprisingly, *S. ransomi* was confirmed in pigs, and a *Strongyloides* sp. closely related to *S. ransomi* and *S. venezuelensis*, typically occurring in rodents, was detected in one of the dog samples. The latter *Strongyloides* species is likely a case of spurious parasite, as most of the dogs around BINP are roaming free and their diet undoubtedly comprises scavenging and/or coprophagy (Goldsmid 1970; Campos et al. 2007). Since there is no known *Strongyloides* transmission between pigs and rodents or primates, the two taxa identified in pigs and a dog are unlikely to pose a threat to either mountain gorillas or humans. However, *S. stercoralis* HVR-IV haplotype E detected in the other dog sample corresponds to the zoonotic *cox1* lineage A occurring in both dogs and humans, although this specific haplotype has so far been reported in dogs only (Beknazarova et al. 2019). Nevertheless, Ashford et al. (1990) reported *S. stercoralis* larvae in humans in Bwindi, therefore the risk for human population is possible. Similarly, although rather rare in free-living NHP (Nosková et al. 2024), S. *stercoralis* may infect the mountain gorillas which can lead to serious health problems, especially in immunocompromised individuals (Penner 1981; Streit 2021).

This study provides the first genetic identification of *Strongyloides* spp. from mountain gorillas, baboons, dogs, and pigs around BINP. Our findings highlight an elevated risk of *Strongyloides* infection among mountain gorillas ranging close to the forest edge, likely due to shared environmental exposure and potential cross-species transmission with sympatric baboons. The detection of *S. stercoralis* Lineage A, a zoonotic genotype, in a local dog underscores the public health implications and raises concerns about possible transmission to humans and endangered great apes.

While previous studies have detected *Strongyloides* spp. around BINP through microscopy, (Ashford et al. 1996; Kalema-Zikusoka et al. 2005; Rothman et al. 2006) the use of molecular diagnostics provides stronger evidence for parasite identity and host overlap. Further research including sampling from people living in the park’s proximity as well as utilization of metagenomics is needed to better understand transmission dynamics and the zoonotic potential of circulating *Strongyloides* strains around BINP. These findings emphasize the importance of adopting One Health-based interventions at the human-wildlife interface. Strategies such as reinforcing park boundaries, limiting the entry of humans and domestic animals, improving sanitation and community awareness are critical to reducing parasite transmission and safeguarding the health of both human populations and the critically endangered mountain gorillas.

## Supplementary Information

Below is the link to the electronic supplementary material.

Supplementary Material 1 (XLSX 11.3 KB)

## Declarations

Fecal samples from animals involved in this study were collected non-invasively without causing any disturbance to the animals. In the case of domestic animals, consent was obtained from the owner. The study was approved by the Veterinary Ethical Review Committee (VERC) REF 1.23 from the University of Edinburgh, Uganda Wildlife Authority (UWA) REF COD/96/05, and the Uganda National Council for Science & Technology (UNCST) REF HS2416ES.

### Data

Sequences obtained in this study have been deposited in the GenBank under the accession numbers PX116718-722 (18S rDNA HVR-IV) and PX116723-729 (*cox1*).
